# Repurposing drugs to treat trichinellosis: *in vitro* analysis of the anthelmintic activity of nifedipine and *Chrysanthemum coronarium* extract

**DOI:** 10.1186/s12906-023-04076-8

**Published:** 2023-07-17

**Authors:** Mona Hasan El-Sayad, Eman Sayed El-Wakil, Zizi Hesham Moharam, Naglaa Fathi Abd El-Latif, Mosad A. Ghareeb, Heba Elhadad

**Affiliations:** 1 Department of Parasitology, Medical Research Institute, Alexandria University, 165 El-Horreya Avenue, El- Hadra (POB: 21561), Alexandria, Egypt; 2 Department of Parasitology, Theodor Bilharz Research Institute, Kornaish El-Nile, Warrak El-Hadar, Imbaba (P.O. 30), Giza, 12411 Egypt; 3 Medical Research Institute, Alexandria University, Alexandria, Egypt; 4 Medicinal Chemistry Department, Theodor Bilharz Research Institute, Kornaish El-Nile, Warrak El-Hadar, Imbaba (P.O. 30), Giza, 12411 Egypt

**Keywords:** *Trichenella spiralis*, Nifedipine, *Chrysanthemum coronarium*, *In vitro* assay, Anthelmintic effect, Scanning electron microscopy, In silico studies

## Abstract

Albendazole is the most common benzimidazole derivative used for trichinellosis treatment but has many drawbacks. The quest for alternative compounds is, therefore, a target for researchers. This work aims to assess the *in vitro* anthelmintic effect of nifedipine, a calcium channel blocker, and a methanol extract of the flowers of *Chrysanthemum coronarium* as therapeutic repurposed drugs for treating different developmental stages of *Trichinella spiralis* in comparison with the reference drug, albendazole. Adult worms and muscle larvae of *Trichinella spiralis* were incubated with different concentrations of the studied drugs. Drug effects were evaluated by parasitological and electron microscopic examination.

As a result, the effects of these drugs on muscle larvae were time and dose-dependent. Moreover, the LC_50_ after 48 h incubation was 81.25 µg/ml for albendazole, 1.24 µg/ml for nifedipine, and 229.48 µg/ml for *C. coronarium*. Also, the effects of the tested drugs were prominent on adult worms as the LC_50_ was 89.77 µg/ml for albendazole, 1.87 µg/ml for nifedipine, and 124.66 µg/ml for *C. coronarium*. SEM examination of the tegument of *T. spiralis* adult worms and larvae showed destruction of the adult worms' tegument in all treated groups. The tegument morphological changes were in the form of marked swellings or whole body collapse with the disappearance of internal contents. Furthermore, in silico studies showed that nifedipine might act as a *T. spiralis* β-tubulin polymerization inhibitor.

Our results suggest that nifedipine and *C. coronarium* extract may be useful therapeutic agents for treating trichinellosis and warrant further assessment in animal disease models.

**Supplementary Information:**

The online version contains supplementary material available at 10.1186/s12906-023-04076-8.

## Introduction

Trichinellosis is a zoonotic parasitic disease caused by nematodes of the genus *Trichinella* [1]. Consuming raw or undercooked pork containing *Trichinella* infective larvae is the transmission route for various mammals [2]. *T. spiralis* is a parasite that completes its biological cycle in the same host; hence it is frequently used as a model to determine the efficacy of various anthelmintic drugs [3].

The primary anthelmintic drugs utilized in the clinical management of trichinellosis are benzimidazole derivatives. However, they have numerous drawbacks [4, 5], as none of these drugs is powerful enough to kill encapsulated and new-born larvae [6] due to their low bioavailability [7], and the development of drug resistance [8]. Likewise, most of them are contraindicated in pregnant women and children below two years of age [3]. Therefore, scientific research aims to discover a novel, secure, and effective anthelmintic agent against *T. spiralis*. In addition, drug repurposing has recently emerged as a tool for developing new indications for existing, failed, or abandoned drugs [9].

Nifedipine is a calcium channel blocker primarily used as an antihypertensive and antianginal medication. It inhibits the transmembrane influx of calcium into cardiac and smooth muscles affecting their contractility [10]. Nematode muscle cells are similar to smooth muscles, with an important distinguishing feature dependent on extracellular calcium for contraction [11]. On the other hand, there is a growing need to find new therapeutic natural agents as a simple replacement for synthetic chemical agents [12, 13]. The *Chrysanthemum* genus belongs to the *Asteraceae* family, with nearly 300 species. It has undergone extensive research on the biological functions and chemical composition of its species. For instance, *C. morifolium*, a popular dietary supplement in China, has been shown to exhibit antihepatotoxic and antigenotoxic effects [14, 15]. Other effects include anti-inflammatory, humoral, and cellular immunomodulatory [16]. Additionally, the flowers were demonstrated to have insecticidal and herbicide effects [17]. Moreover, *C. coronarium* has been tried as an antibacterial agent with promising results [18]. This study aims to assess the in vitro effects of nifedipine, a calcium channel blocker, and a methanol extract of flowers of *C. coronarium* as anthelminthic agents for different developmental stages of *T. spiralis* in comparison with the reference drug, albendazole.

## Materials and methods

### Parasite

*Trichinella spiralis* was kindly provided by the Parasitology Department, Theodor Bilharz Research Institute (TBRI), Giza, Egypt, from laboratory-bred infected Swiss albino mice. Mice were orally infected with 200–300 T*. spiralis* larvae and maintained following the institutional and national guidelines in the animal house exposed to 12 h light/12 h dark cycles with a free access to standard pelleted diet) Delta group, Egypt) and water ad libitum [19].

### Isolation of adult worms and muscle larvae

The collection of adult worms was typically performed at five days post-infection (dpi). Ten infected mice were sacrificed under light anesthesia by isoflurane inhalation (Forane®, UK). Then, the intestines of infected mice were removed and opened with scissors. The opened intestines were gently washed in phosphate-buffered saline (PBS) to remove the intestinal contents. Afterward, the small intestines were cut into 2 cm sections, slit longitudinally, and placed on a gauze in a beaker containing 250 ml of PBS for 3 h at 37 °C [20].

In addition, muscle larvae (ML) were obtained from infected mice on day 35 post-infection. Then, each mouse was dissected, and the muscles were digested in 200 ml of distilled water with 1% concentrated HCl and 1% pepsin. The mixture was continuously stirred with an electric stirrer for two hours at 37 °C. On a 200-mesh/inch screen, the larvae released from cysts were collected, washed twice in tap water, and then suspended in a conical flask in 150 ml of tap water. Finally, the supernatant fluid was removed, and a McMaster counting chamber was used to count the larvae in the sediment under a microscope [21].

### Extraction and preparation of *C. coronarium*

*Chrysanthemum coronarium* fresh flowers were collected from El-Fayoum Governorate, Egypt, in January 2021. Botany specialists from the Department of Flora and Taxonomy, Faculty of Science, Alexandria, Egypt, established the identification and authentication of the collected plant. A voucher specimen number (C.c.f.2021) was deposited in the Medicinal Chemistry Department, TBRI. *C. coronarium* air-dried flowers (0.5 kg) were extracted four times via maceration using methanol (4L) at room temperature (22–26 ºC). The extract was concentrated via a rotary evaporator (BUCHI R-300, Switzerland) at 40 ºC to obtain 46 g of methanol extract. The extraction yield was equal to 9.2% calculated by the following equation: Yield (%) = Dry extract weight/ dry powder weight × 100 [22].

### Identification of the binding site of nifedipine to *T. spiralis* β-tubulin

#### Template-based model construction

The FASTA formatted amino acid sequence of *T. spiralis* β-tubulin was obtained from the NCBI GenBank database (GenBank: EFV50889) [23]. The I-TASSER service (http://zhanglab.ccmb.med.umich.edu/I-TASSER/) was used to identify binding sites and templates for this sequence [24]. Due to the presence of amino acids associated with nematode resistance in the binding site discovered by I-TASSER, the D chain, which is present in the b-subunit of the heterotetrameric structure of *Ovis aries* (PDB ID: 3N2G D), was selected as a template [25]. *T. spiralis* β-tubulin residues 1–428 were used in the homology modeling method conducted with MODELLER 9v10 software [26].

#### Deep learning-based model construction

The Alphafold-2 deep-learning-based technique was used to create another model for *T. spiralis* β-tubulin [27]. Then, ten models were created, and the one with the lowest energy was selected for optimization. After that, a series of molecular dynamics simulations were carried out to refine the template-based and Alphafold-2-based models.

#### Molecular dynamic simulation

We essentially followed the iterative technique that had previously been reported. We simulated each generated model for 50 ns at 310 K, and ran five different trajectories. During the scoring process, we utilized RWplus [28].

#### Docking Study

The modeled structure of nifedipine was docked into the predetermined binding site in model-generated homology modeling using AutoDock Vina. The docking grid-box's coordinates were set to be: x = 115.43, y = 90.11, z = 7.90. The ligand to binding site shape matching root means square (RMSD) threshold was set to 2.0 Å. The interaction energies were determined using the Charmm force field (v.1.02) with 10.0 Å as a non-bonded cutoff distance and distance-dependent dielectric. Then, 5.0 Å was set as an energy grid extending from the binding site. The tested compound retinol was energy minimized inside the selected binding pocket. The editing and visualization of the generated binding poses were performed using PyMOL software [29].

### Drug concentrations

Different concentrations of albendazole, nifedipine, and *C. coronarium* extract were investigated. Albendazole (Pharco Pharmaceuticals, Egypt) stock solution was prepared in 1% dimethyl sulfoxide (DMSO) at 400 µg /ml concentration. Then, serial dilutions were carried out to obtain 200, 100, 50, and 25 µg/ml concentrations [30]. Different concentrations of nifedipine (25, 12.5, 6.25, 3.125, 1.56, 0.78 µg/ml) were prepared from Epilat capsules (Epico, Egypt). Next, *C. coronarium* (400 µg/ml) was prepared by dissolving 400 µg of the extract into one ml of distilled water. After that, serial dilutions were conducted from this preparation to obtain 200, 100, 50, and 25 µg/ml concentrations.

### Experimental design

All experiments were carried out in a sterile 24-well tissue culture plate (SoCal BioMed, USA). Fifty muscle larvae or 25 adult worms were incubated in 2 ml of Rapid Prototyping and Manufacturing Institute medium (RPMI)-1640 (Lonza, Belgium) containing 10% fetal calf serum, 200 U/ml penicillin, 200 μg/ml streptomycin (Omega Scientific, USA), and the desired drug concentration [31]. Negative control (blank) containing only the parasite (adult worms or ML) in pure culture media or DMSO control were included in all assays and subjected to the same conditions as the experimental cultures. Then, the plates were sealed and incubated at 37 °C in an atmosphere containing 5% CO_2_ for 1, 6, 24, 48, and 72 h. The observations were prolonged to 96 h for the ML. The experiments were carried out in duplicates, and data were compared with the DMSO control for albendazole and the corresponding blank control for nifedipine and *C. coronarium*. The efficacy of the studied drugs was evaluated by parasitological and scanning electron microscope examination [19].

### Parasitological studies

The viability of *Trichinella* stages cultured in vitro in different concentrations was evaluated by assessing their shapes and mobility. They were counted either alive or dead. The average value of the two experiments was calculated [19].

### Scanning electron microscope (SEM) examination

*T. spiralis* adult worms or ML were directly pipetted into a fresh fixation solution of 2.5% glutaraldehyde (w/v) in 0.1 M sodium cacodylate at pH 7.2 and left overnight at 37 °C. *Trichinella* stages were washed in 0.1 M sodium cacodylate buffer at pH 7.2 for 5 min, post-fixed in a 2% (w/v) osmium tetroxide in sodium cacodylate buffer for one hour. The specimens were dehydrated in an ethanol series and dried using liquid carbon dioxide. The dried parasite stages were sprinkled onto and mounted on stubs bearing double sided carbon adhesive tape. Then, samples were coated with gold using sputter coater (EIKO Engineering CO, Japan), and examined by SEM (Jeol Corp., Japan) at 5 to 20 kV in the scanning electron microscopy lab, at the Faculty of Science, Alexandria University, Egypt [32].

### Statistical analysis

Data were presented as the mean ± standard deviation (S.D.). The results were analyzed using IBM SPSS software package version 20.0 (IBM Corp, USA) [33]. The student's t-test analyzed the significance of the differences between the experimental and the control groups. Moreover, the one way ANOVA and post hoc Tuky HSD with Bonferroni correction tests were applied for multigroup comparisons using the Rstatix package in Rstudio (R version 4.2.3). *P*-value < 0.05 was significant, and *P*-value < 0.001 was highly significant. All graphs were generated by the ggplot2 package. The half-lethal concentration **(**LC_50_) at 48 h after incubation with different drugs was calculated based on the Quest Graph online program https://www.aatbio.com/tools/ic50-calculator.

## Results

### Homology modelling and binding site prediction

**Fig. 1:**
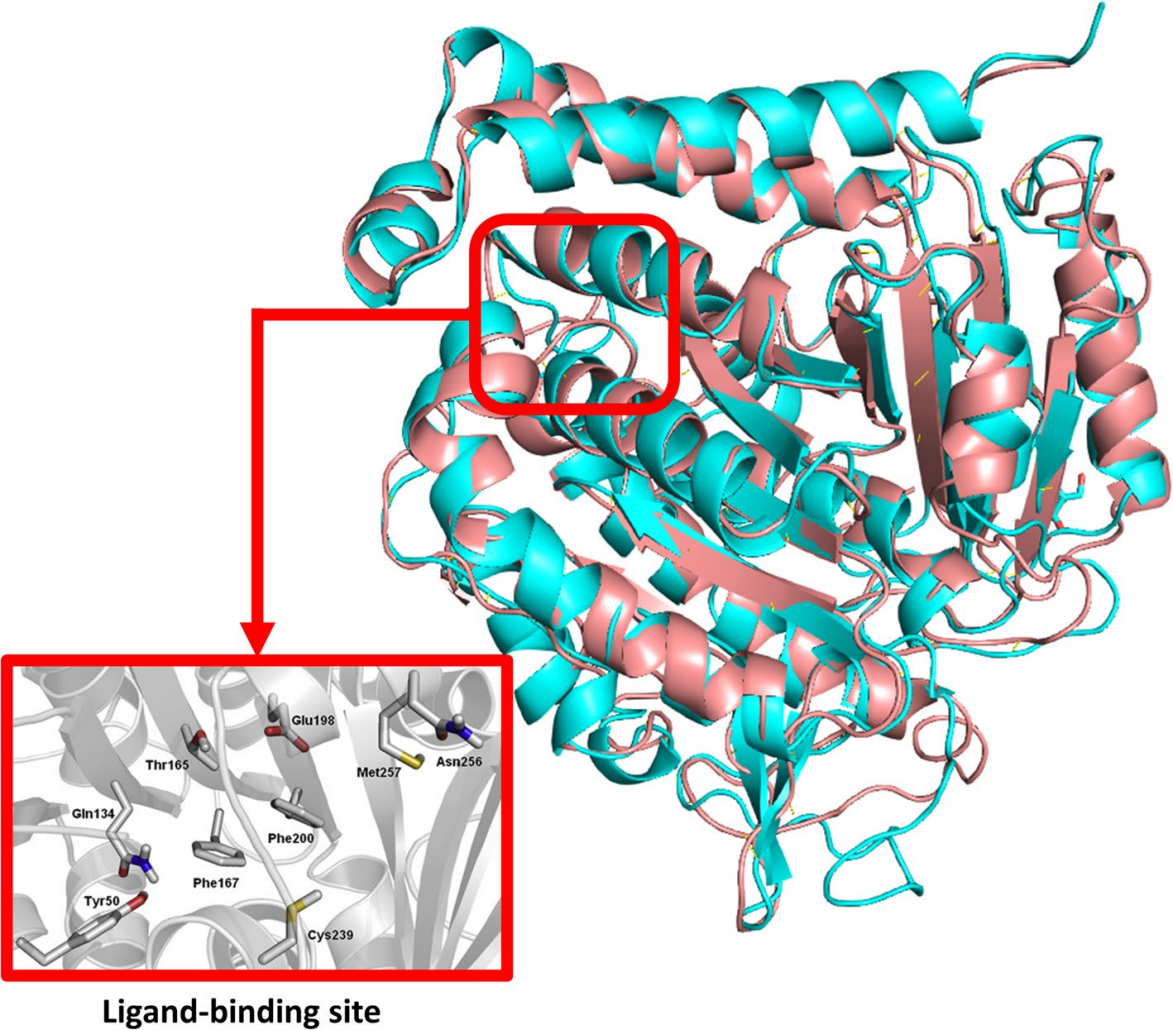
Structural alignment of two generated *T. spiralis* β-tubulin models showing the ligand-binding site. The cyan-colored structure is the Alphafold2-generated model, and the brick-red-colored structure is the template-generated model (RMSD = 1.16 Å)

Due to the lack of a crystallographic structure for *T. spiralis* β-tubulin, we generated a homology model by mining the known repertoire of helminth resistance mutations for a suitable template [34]. The structure of *T. spiralis* β-tubulin was generated by the *O. aries* β-tubulin (PDB ID: 3N2G; D subunit) as a starting point. There were 406 completely identical places and 18 partially identical positions aligned with the template and target sequences. With the I-TASSER platform, MODELLER software, and the PDB ID: 3N2G; D subunit as a starting point, we could create a 3D model of the *T. spiralis* β-tubulin (Fig. 1).

**Fig. 2:**
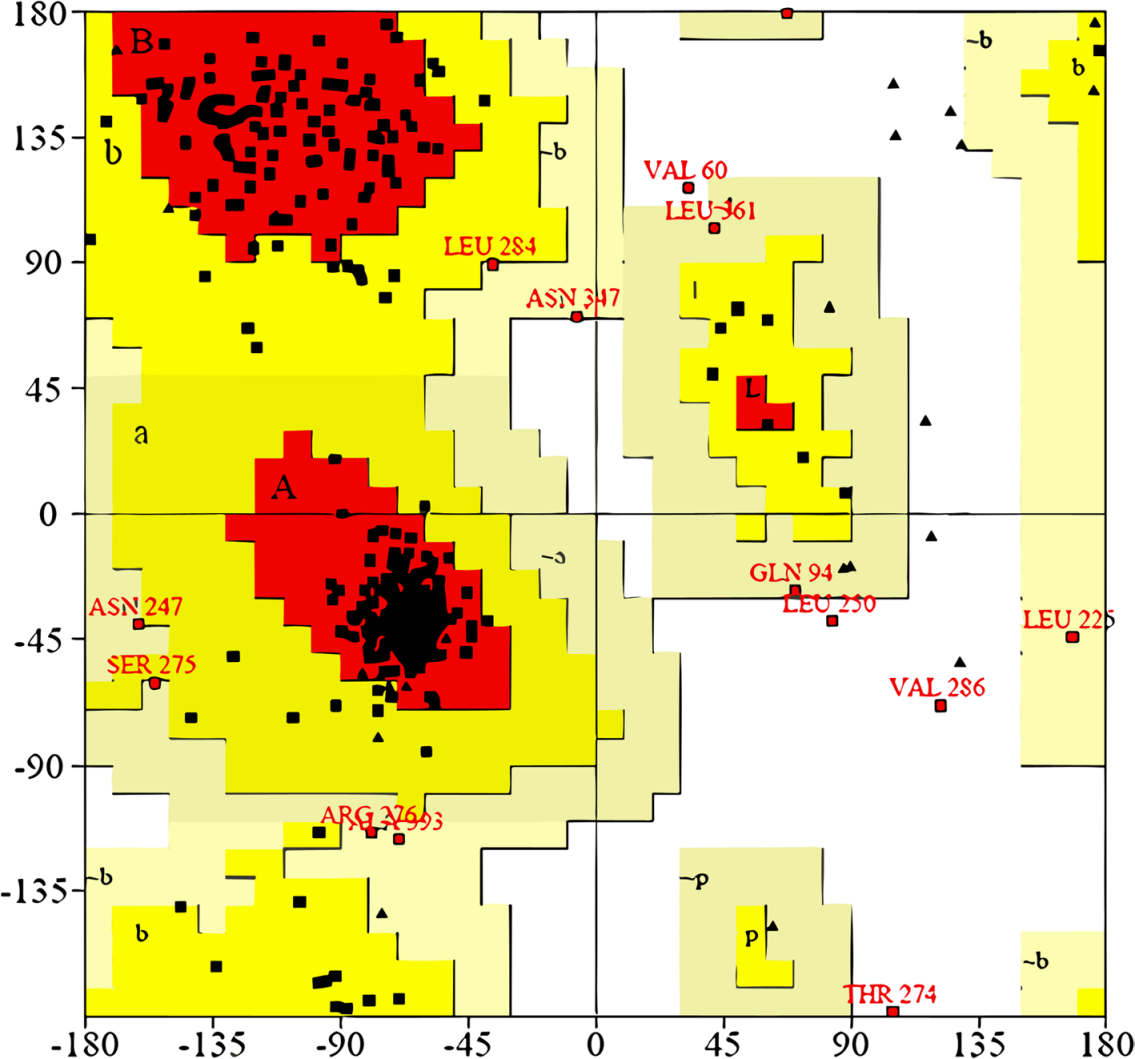
The Ramachandran plot highlights the amino acids' preferred and forbidden zones

For more confirmation, the β-tubulin sequence was also subjected to Alphafold-2 to build another 3D model using a different method (i.e., deep neural networking-based de novo model construction) [2]. The resulting best-scoring and the first template-based models were subjected to 50 ns-long M.D simulations to relax the generated structures. In addition, the most populated structures for each model from each trajectory were extracted and compared. Upon alignment of the generated structures to each other, they were almost identical (RMSD = 1.16 Å). The Ramachandran plot analysis showed a reliability of 96.7% and placed all the amino acids corresponding to the co-crystallized ligand's binding site inside the permissible zones (Fig. 2). This model is high quality enough to be used in molecular docking. Figure 1 reveals that Phe167, Glu198, and Phe200, three of the most significant amino acids in helminth resistance, form the binding pocket [1]. Near the monomer–monomer interface of the heterodimer (in the N-terminal domain of the B monomer), the proposed binding site in β-tubulin models consists of several highly conserved hydrophobic amino acids (Leu240, Leu250, Leu253, and Phe266), and a few hydrophilic residues (Thr237, and Asn256).

### Molecular docking

To investigate their binding modes, the *O. aries* β-tubulin co-crystallized inhibitor and nifedipine structures were docked into the generated *T. spiralis* β-tubulin model (i.e., the template-based one). The structure of colchicine, a well-known β-tubulin inhibitor, was also docked as a reference inhibitor. The three ligands got comparable docking scores (-7.67, -7.54, and -7.41 kcal/mol, respectively).

**Fig. 3:**
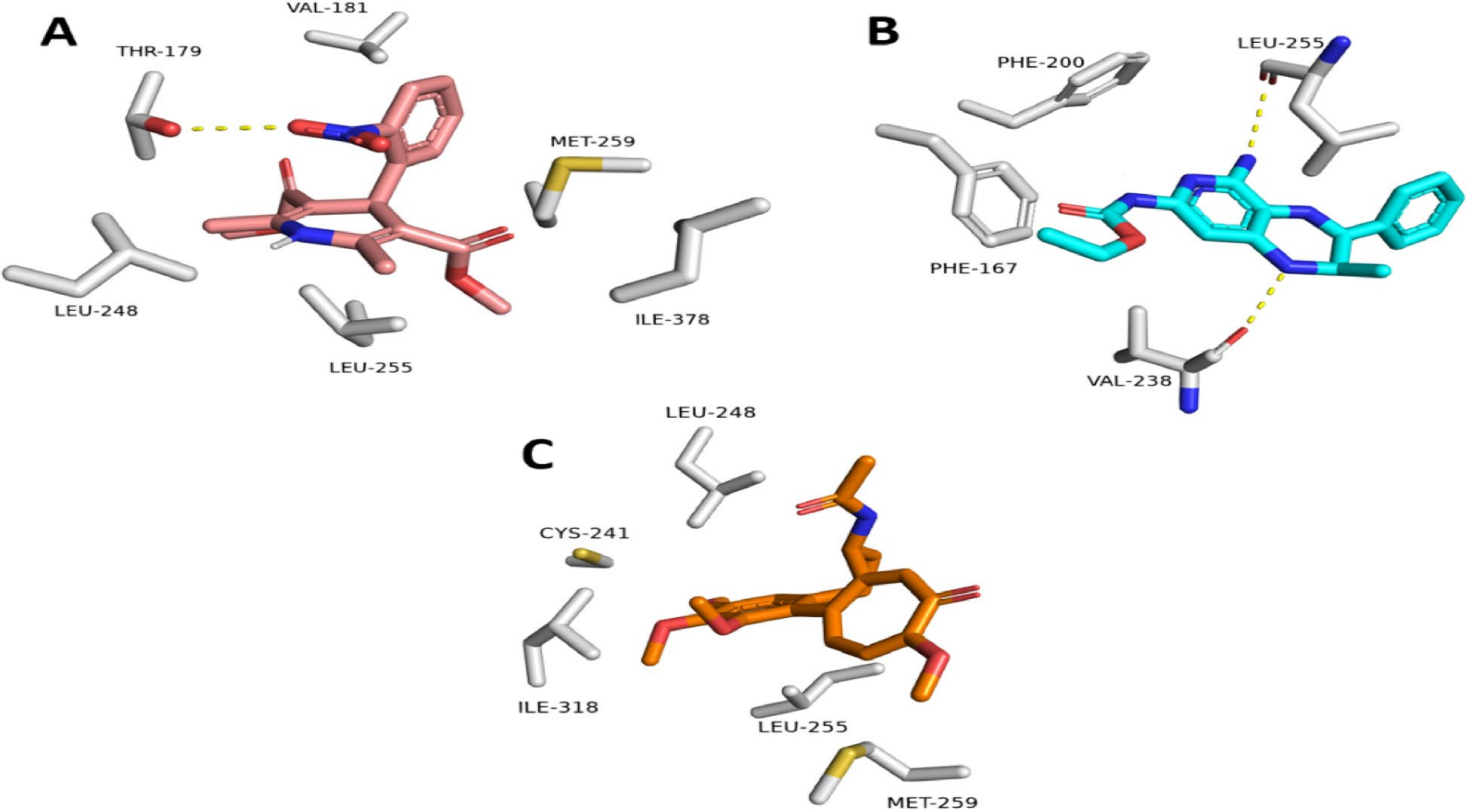
Binding modes of nifedipine inside the *T. spiralis* β-tubulin's binding site alongside the two reference inhibitors (i.e., *O. aries* β-tubulin co-crystallized inhibitor and colchicine) (**A**-**C**, respectively)

Figure 3 shows that hydrophobic interactions are the key to the three ligands. The binding mode of the nifedipine structure was more convergent to that of the colchicine than that of the *O. aries* β-tubulin co-crystallized inhibitor, where they established hydrophobic interactions with LEU-248, LEU-255, MET-259 (Figs. 3A and C). Accordingly, it can be concluded that nifedipine might act as a *T. spiralis* β-tubulin polymerization inhibitor.

### The effect of the studied drugs

The studied drugs showed a remarkable parasiticidal effect on *T. spiralis*. The effects on muscle larvae are shown in Table 1. At 25 µg/ml, albendazole showed a statistically significant effect after 96 h. It also killed all larvae after 48 h in concentrations starting at 100 µg/ml. The statistically significant larvicidal effect was evident at a concentration of 100 µg/ml and above from the first hour of exposure. Moreover, the LC_50_ of albendazole was calculated to be 81.25 µg/ml. As regards nifedipine, a statistically significant larvicidal effect was obtained at a concentration of 0.78 µg/ml after 4 h. In addition, it killed all larvae after 72 h in concentrations starting from 3.125 µg/ml. The LC_50_ of nifedipine was calculated to be 1.24 µg/ml. *C*. *coronarium* killed 100% of larvae at a high concentration (400 µg/ml) after 96 h and it needed at least 48 h to exert its effect on incubated ML. A statistically significant difference was recorded initially at 200 µg/ml after 48 h (*P* ≤ 0.05). Then, the LC_50_ was calculated to be 229.48 µg/ml.

**Table 1:** Survival numbers of *T. spiralis* muscle larvae incubated with different concentrations of studied drugs (*n* = 50)

Drugs	Dose µg/ml	**Exposure Times (h.)**						ANOVA *p*	LC_50_
		1 h	4 h	24 h	48 h	72 h	96 h		
Blank control		50.0 ± 0.0	48.5 ± 2.1	44.0 ± 1.4	39.5 ± 3.5	35.0 ± 2.8	26.0 ± 1.4		
DMSO control		48.5 ± 2.1	44.5 ± 2.1	40.5 ± 0.7	36.0 ± 1.4	30.0 ± 0.0	22.0 ± 1.4		
**Albendazole**	25	47.0 ± 2.83	45.5 ± 0.71	41.5 ± 0.71	34.0 ± 1.41	28.0 ± 1.41	19.5 ± 0.71^*^	**0.013**	81.25 µg/ml
	50	46.0 ± 1.41	43.0 ± 0.00^*^	36.0 ± 1.41^*^	28.0 ± 1.41^**^	16.0 ± 1.41^**^	10.0 ± 1.41^**^	** < 0.001**	
	100	40.5 ± 0.71^*^	36.0 ± 1.41^**^	19.0 ± 0.00^**^	2.5 ± 0.71^**^	0.0 ± 0.00^**^	0.0 ± 0.00^**^	** < 0.001**	
	200	33.5 ± 2.12^**^	16.0 ± 1.41^**^	7.0 ± 0.00^**^	0.0 ± 0.00^**^	0.0 ± 0.00^**^	0.0 ± 0.00^**^	** < 0.001**	
	400	22.5 ± 3.54^**^	10.0 ± 1.41^**^	2.5 ± 3.54^**^	0.0 ± 0.00^**^	0.0 ± 0.00^**^	0.0 ± 0.00^**^	** < 0.001**	
ANOVA *p*		** < 0.001**	** < 0.001**	** < 0.001**	** < 0.001**	** < 0.001**	** < 0.001**		
**Nifedipine**	0.78	46.0 ± 1.41	39.5 ± 0.71^**^	39.0 ± 2.83	37.0 ± 2.83	31.0 ± 0.00	20.5 ± 2.12	0.134	1.24 µg/ml
	1.56	42.0 ± 1.41^*^	34.0 ± 1.41^**^	26.0 ± 1.41^**^	22.0 ± 1.41^**^	19.0 ± 0.00^**^	14.0 ± 2.83	**0.002**	
	3.125	38.5 ± 2.12^*^	26.5 ± 2.12^**^	20.0 ± 1.41^**^	10.5 ± 0.71^**^	0.0 ± 0.00^**^	0.0 ± 0.00^**^	** < 0.001**	
	6.25	35.0 ± 2.83^**^	20.0 ± 1.41^**^	13.0 ± 2.83^**^	3.0 ± 0.00^**^	0.0 ± 0.00^**^	0.0 ± 0.00^**^	** < 0.001**	
	12.5	30.0 ± 1.41^**^	15.0 ± 2.83^**^	8.5 ± 0.71^**^	0.0 ± 0.00^**^	0.0 ± 0.00^**^	0.0 ± 0.00^**^	** < 0.001**	
ANOVA *p*		**0.0026**	** < 0.001**	** < 0.001**	** < 0.0001**	** < 0.0001**	** < 0.0001**		
***C. coronarium***	25	50.0 ± 0.00	48.5 ± 0.71	46.5 ± 0.71	40.5 ± 0.71	34.0 ± 1.41	24.0 ± 1.41	0.081	229.48 µg/ml
	50	49.0 ± 0.00	48.5 ± 0.71	44.5 ± 0.71	40.0 ± 1.41	29.5 ± 3.54	21.0 ± 1.41	**0.007**	
	100	47.0 ± 0.00	45.0 ± 0.00	42.0 ± 1.41	36.5 ± 2.12	23.0 ± 2.83	15.0 ± 2.83	**0.001**	
	200	47.0 ± 0.00	44.5 ± 0.71	40.5 ± 0.71	29.0 ± 2.83^*^	16.0 ± 1.41^**^	7.5 ± 2.12^**^	** < 0.001**	
	400	47.0 ± 2.83	42.0 ± 1.41	38.0 ± 1.41	20.0 ± 1.41^**^	9.0 ± 2.83^**^	0.0 ± 0.00^**^	** < 0.001**	
ANOVA *p*		0.171	**0.002**	**0.003**	** < 0.001**	** < 0.001**	** < 0.001**		

The muscle larvae survival was represented as Mean ± S.D

Student's t-test was used to compare data with the corresponding control

^*^P value ≤ 0.05 significant; **P value ≤ 0.01 highly significant

ANOVA test was used for multigroup comparison regarding the different concentrations and incubation periods

Bold *p*-values indicate significant differences between groups at alpha level < 0.05

LC_50_ at 48 h was calculated based on The Quest Graph online program https://www.aatbio.com/tools/ic50-calculator

Table 2 shows the effects of the tested drugs on adult worms. At low doses of 25 and 50 µg/ml, albendazole showed a non-statistically significant effect even after 72 h. The statistically significant effect was evident starting from 4 h of exposure at 100 µg/ml (*P* ≤ 0.05) or higher concentrations (*P* ≤ 0.01). Albendazole killed all worms after 24 h in concentrations starting from 200 µg/ml. The LC_50_ of albendazole was calculated to be 89.77 µg/ml. Regarding nifedipine, a statistically significant effect was obtained at a concentration of 1.56 µg/ml after 4 h (*P* ≤ 0.05). Nifedipine killed all worms after 4 h in concentration starting from 6.25 µg/ml. The LC_50_ of nifedipine was calculated to be 1.87 µg/ml. *C. coronarium* caused the death of all adult worms at high concentrations (200 µg/ml and 400 µg/ml) after 72 h. A statistically significant difference was recorded initially after incubation at 100 µg/ml for 72 h (*P* ≤ 0.05). The LC_50_ of *C. coronarium* was calculated to be 124.66 µg/ml.

**Table 2:** Survival numbers of *T. spiralis* adult worms incubated with different concentrations of studied drugs (*n* = 25)

Drugs	Dose µg/ml	**Exposure Times (h)**					ANOVA *p*	LC_50_ µg/ml
		1 h	4 h	24 h	48 h	72 h		
Blank control		25.0 ± 0.0	23.5 ± 0.7	21.0 ± 1.4	18.0 ± 1.4	14.5 ± 0.7		
DMSO control		23.5 ± 0.7	21.0 ± 1.4	19.0 ± 1.4	16.5 ± 2.1	11.0 ± 2.8		
**Albendazole**	25	24.5 ± 0.71	22.5 ± 0.71	19.5 ± 0.71	16.5 ± 2.12	15.0 ± 2.83	0.698	89.77 µg/ml
	50	22.0 ± 1.41	21.0 ± 1.41	16.0 ± 1.41	13.5 ± 2.12	11.5 ± 0.71	**0.017**	
	100	22.0 ± 1.41	16.5 ± 2.12^*^	6.5 ± 3.54^**^	2.0 ± 0.00^**^	0.0 ± 0.00^**^	**0.002**	
	200	10.5 ± 0.71^**^	8.0 ± 1.41^**^	0.0 ± 0.00^**^	0.0 ± 0.00^**^	0.0 ± 0.00^**^	** < 0.001**	
	400	4.0 ± 1.41^**^	0.0 ± 0.00^**^	0.0 ± 0.00^**^	0.0 ± 0.00^**^	0.0 ± 0.00^**^	**0.01**	
ANOVA *p*		** < 0.0001**	** < 0.0001**	** < 0.001**	** < 0.001**	** < 0.001**		
**Nifedipine**	0.78	24.0 ± 1.41	22.5 ± 0.71	19.5 ± 2.12	17.0 ± 2.83	14.0 ± 4.24	0.998	1.87 µg/ml
	1.56	21.5 ± 2.12	17.5 ± 2.1^*^	12.5 ± 2.12^*^	10.0 ± 1.41^*^	7.5 ± 3.54^*^	**0.031**	
	3.125	11.5 ± 0.71^**^	6.0 ± 1.41^**^	2.0 ± 1.41^**^	0.0 ± 0.00^**^	0.0 ± 0.00^**^	** < 0.001**	
	6.25	10.5 ± 0.71^**^	0.0 ± 0.00^**^	0.0 ± 0.00^**^	0.0 ± 0.00^**^	0.0 ± 0.00^**^	** < 0.001**	
	12.5	6.0 ± 1.41^**^	0.0 ± 0.00^**^	0.0 ± 0.00^**^	0.0 ± 0.00^**^	0.0 ± 0.00^**^	**0.002**	
ANOVA *p*		** < 0.001**	** < 0.0001**	** < 0.001**	** < 0.001**	**0.007**		
***C. coronarium***	25	25.0 ± 0.00	24.0 ± 1.41	21.0 ± 0.00	19.5 ± 0.71	16.5 ± 2.12	0.417	124.66 µg/ml
	50	25.0 ± 0.00	23.5 ± 0.71	19.5 ± 0.71	16.5 ± 0.71	13.0 ± 2.83	0.758	
	100	23.0 ± 0.00	22.0 ± 0.00	18.0 ± 1.41	13.0 ± 2.83	5.0 ± 2.83^*^	**0.011**	
	200	22.0 ± 1.41	20.5 ± 0.71	15.5 ± 2.12^*^	5.5 ± 3.54^**^	0.0 ± 0.00^**^	** < 0.001**	
	400	19.5 ± 0.71^*^	17.0 ± 2.8^*^	8.0 ± 1.41^**^	2.5 ± 0.71^**^	0.0 ± 0.00^**^	** < 0.001**	
ANOVA *p*		**0.002**	**0.026**	**0.001**	**0.001**	**0.001**		

The survival of adult worms was represented as Mean ± S.D

Student's t-test was used to compare data with the corresponding control

^*^P value ≤ 0.05 significant, **P value ≤ 0.01 highly significant

ANOVA test was used for multigroup comparison regarding the different concentrations and incubation periods

Bold *p*-values indicate significant differences between groups at alpha level < 0.05

LC_50_ at 48 h was calculated based on The Quest Graph online program https://www.aatbio.com/tools/ic50-calculator

**Fig. 4:**
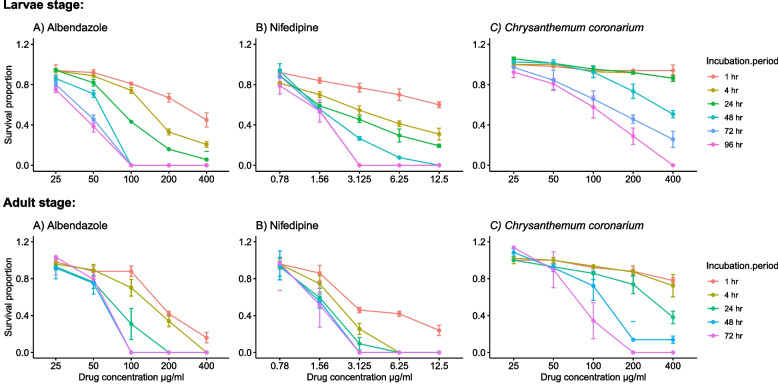
Proportions of *T. spiralis* ML and adult worms survival at the different concentrations of the studied drugs. Data normalization relative to the corresponding control was performed

**Fig. 5:**
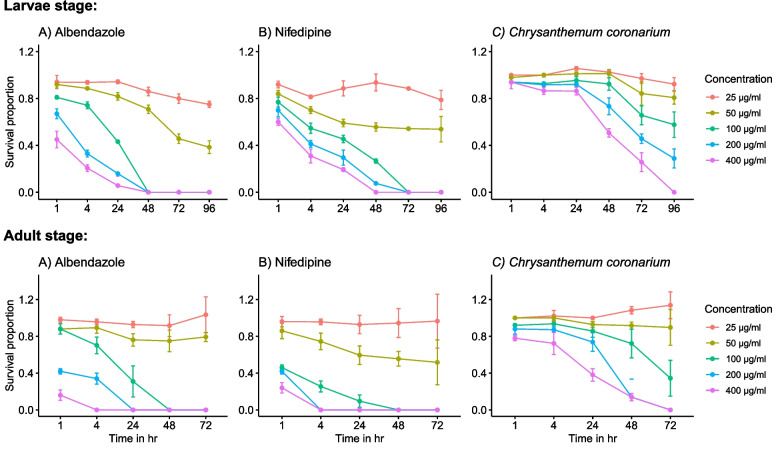
Proportions of *T. spiralis* ML and adult worms survival at different incubation times with studied drugs. Data normalization relative to the corresponding control was performed

The effects of the studied drugs on muscle larvae and adult worms were dose (Fig. 4) and time (Fig. 5) dependant. However, incubating muscle larvae with different concentrations of *C. coronarium* does not affect their survival in the first 24 h. There was no significant difference between blank and DMSO controls at all test incubation periods. A comparison of the newly tried drugs, nifedipine and *C. coronarium* with the reference drug, albendazole at the closest concentrations to the calculated LC_50_ is shown in (Table 3). Statistically significant differences were recorded between the studied drugs and albendazole on muscle larvae and adult worms.

**Table 3:** The effect of nifedipine and *C. coronarium* on *T. spiralis* developmental stages compared to the reference drug, albendazole, 48 h after incubation

Drugs	**Albendazole** (100 µg/ml)	**Nifedipine** (1.56 µg/ml)	***C. coronarium*** (200 µg/ml)	**F**	*P-value
**Muscle larvae**					
Mean ± SD	2.5 ± 0.71	22.0 ± 1.41	29.0 ± 2.83	**137.4**	**0.001**
Post hoc		*p*1 = **0.0025**	*p*2 = **0.0012**		
			*p*3 = 0.0623		
**Adult worms**					
Mean ± SD	2.0 ± 0.00	10.0 ± 1.41	5.5 ± 3.54	**11.207**	**0.041**
Post hoc		*p*1 = **0.04**	*p*2 = 0.559		
			*p*3 = 0.083		

F statistic & *P obtained by ANOVA test

*p*1: *p*3 obtained by Tukey HSD post hoc test with Bonferroni correction

*P1* comparing albendazole and nifedipine, *p2* comparing albendazole and coronium while, *p3* comparing nifedipine and coronium

Bold *p*-values indicate significant differences between groups at alpha level < 0.05

### Scanning electron microscope findings

**Fig. 6:**
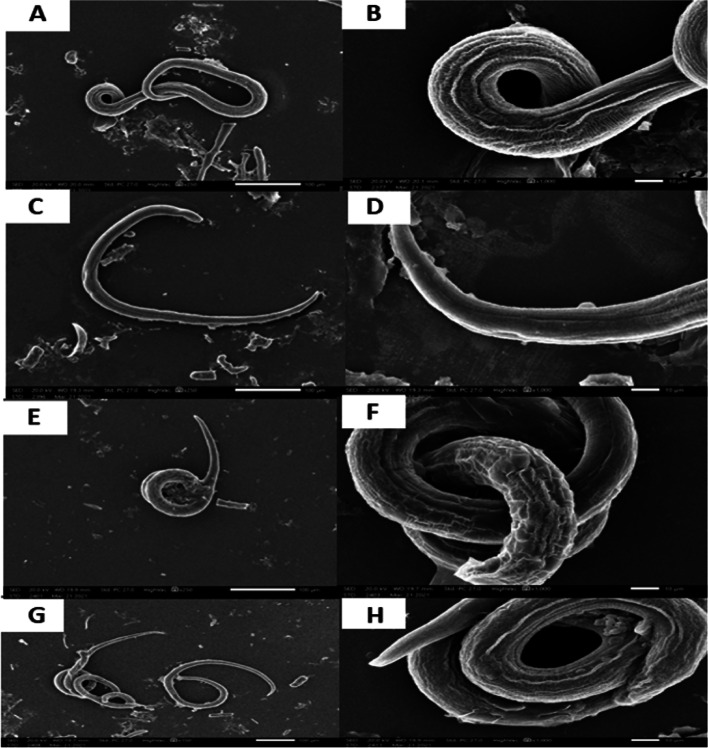
SEM findings of the cultured *T. spiralis* muscle larvae after 48 h incubation. **A, B:** blank control group showing normal cuticle with transverse creases and longitudinal ridges, **C, D:** albendazole (100 µg/ml) treated group, **E,F:** nifedipine (1.56 µg/ml) treated group, **G, H:** *C. coronarium* extract (200 µg/ml) treated group

SEM examination of the tegument of *T. spiralis* ML from the control groups showed the typical coiling behaviour and the characteristic pattern of normal tegument with longitudinal ridges and transverse creases. Conversely, tegument destruction in the treated groups was apparent since multiple degenerative changes, such as the appearance of blebs, multiple vesicles, and loss of normal annulation (Fig. 6). This destruction was especially evident in the subgroup treated with albendazole.

**Fig. 7:**
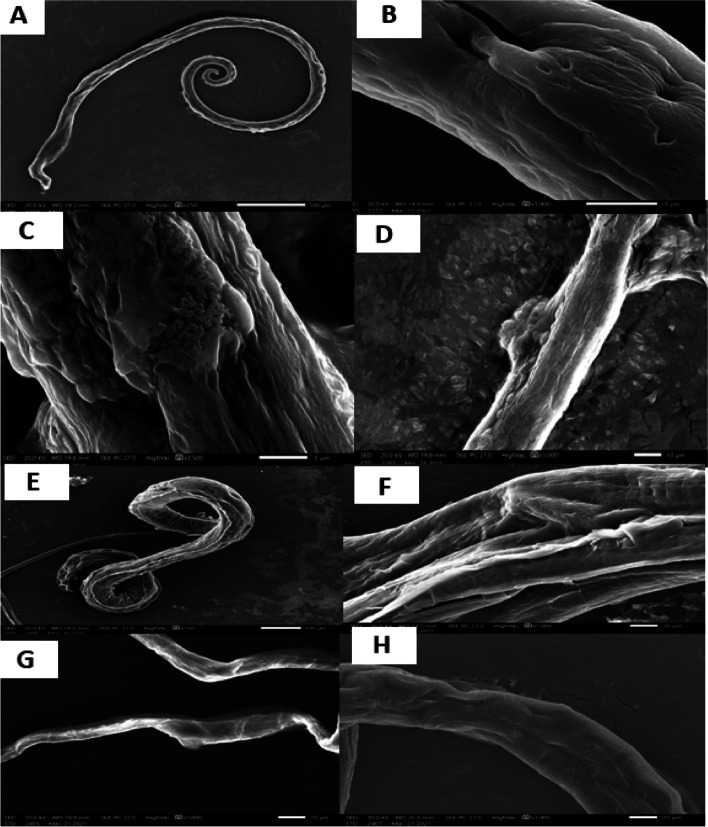
SEM findings of the cultured *T. spiralis* adult worms after 48 h incubation. **A, B:** blank control group showing normal adult worm cuticle with hypodermal glands openings and tapering end, **C, D:** albendazole (100 µg/ml) treated group, **E,F:** nifedipine (1.56 µg/ml) treated group, **G, H:** *C. coronarium* extract (200 µg/ml) treated group

Examination of adult worms by the SEM revealed that the tegument of *T. spiralis* adult worms from the control group showed normal cuticles with hypodermal glands opening and tapering ends. In contrast, the incubation of adult worms with either albendazole or nifedipine caused marked destruction of the adult worms. The tegument showed areas with marked swellings, multiple large blebs, fissures, and vesicles. In addition, sloughing of some areas of the tegument was observed. *C. coronarium* treated groups showed body collapse as well as the appearance of areas of decreased opacity (Fig. 7).

## Discussion

*T. spiralis* is a worldwide nematode infecting various mammalian hosts, including humans, with possible serious complications [35]. The limitations of benzimidazole derivatives to treat the parasite's encapsulated larval stages, in addition to the negative drug reactions, including mortality, encephalitis, convulsions, and severe drug eruptions, have motivated medical researchers to discover novel, safe, and efficient anthelminthic agents [2, 36, 37]. Repurposing the currently available drugs and the use of medicinal plants have emerged as two powerful strategies in the search for new treatment options.

In the present study, albendazole was used as the reference drug for the treatment of *T. spiralis*. The effect of albendazole was the strongest compared to other drugs. LC_50_ was nearly similar between muscle larvae and adults (104.21 µg/ml vs. 96.03 µg/ml). However, higher LC_50_ value against adult worms was reported by Kaiser et al. (> 200 µg/ml) against the adult worms. Also, Priotti et al. studied the effect of albendazole on adult worms and reported that worms' viability was 72% 48 h after incubation with albendazole.

Nifedipine is a potent calcium channel antagonist used to treat hypertension by blocking calcium input into endothelial cells, causing intense relaxation [38]. Nifedipine gained attention due to the discovery of its anti-parasitic properties, mainly against *Plasmodium* spp. and also against other parasites such as *Schistosoma*, *Leishmania,* and *Microsporidia* [39–42]. The results of the present in silico studies indicate that nifedipine might act as a *T. spiralis* β-tubulin polymerization inhibitor. Moreover, it has a lethal activity against muscle larvae and apostdult stages of *T. spiralis in vitro*. This remarkable effect was found to occur in a dose and time-dependent manner. The adult worms seemed more susceptible than muscle larvae since no viable adult worms were found after 4 h incubation with nifedipine at a concentration of 6.25 μg/ml. Similarly, Silva-Moraes et al. evaluated the effect of nifedipine on schistosomula, and adult worms cultures to provide new therapeutic strategies for *Schistosoma mansoni* treatment. Results displayed a significant antischistosomal effect even on the initial life cycle stages of the parasite [39]. Also, a previous study has evaluated the effect of the same drug on the filarial nematode; *Acanthocheilonema viteae,* and reported that it reduces calcium influx across the muscle membrane [43].

Nowadays, there is a broad consensus that diverse plant-derived products have inhibitory effects on many infectious agents. The chemical heterogeneity of the *C. coronarium*'s composition was thought to be responsible for its numerous biological activities [44–46]. Though it has been shown to exhibit antibacterial, antifungal, and antiviral properties [18, 47, 48], not much is known about its antiparasitic activity. In the present study, *C. coronarium* showed a potentially lethal activity against *T. spiralis* muscle larvae and adult worms *in vitro*. The adults were more susceptible than the muscle larvae, as a lower LC_50_ after 48 h incubation was computed (124.66 µg/ml vs. 229.48 µg/ml). These results are in agreement with Bar-Eyal et al. [49], who found that adding *C. coronarium* to the soil as a green manure to suppress the root-knot nematodes *Meloidogyne incognita* and *M. javanica* was fatal. Other studies assessed the antibacterial properties of *C. coronarium* essential oil [48, 50]. These investigations showed that the essential oil has antibacterial activity against Gram-positive bacteria but not against Gram-negative bacteria, which goes in hand with previous literature reports that Gram-positive bacteria are more responsive to essential oil therapies than Gram-negative bacteria [51, 52].

Changes in the tegument of helminths are considered a good indicator for the possible anthelmintic activity of a drug [53]. Blebbing occurs as the parasite attempts to replace the damaged surface membrane in response to drug action. Albendazole disrupts the parasite's metabolic pathways, resulting in diminished ATP production, interfering with cell motility and maintaining cell shape [54, 55] In the present study, the ultra-structural effects of the studied drugs on *T. spiralis* were evident. Similar results were reported by Fahmy et al. [56], who applied clove oil *(Syzygium aromaticum)* against adults and muscle larvae of *T. spiralis*.

As far as we know, this is the first report presenting the results of the anthelmintic activity of nifedipine and *C. coronarium* against *T. spiralis*. Both drugs had remarkable lethal effects on adult forms and muscle larvae. However, one limitation of this study is that in vivo experiments are more useful than *in vitro* experiments, as many *in vitro* active drugs are inactive in living organisms. Therefore, further *in vitro* and in vivo studies should be performed to confirm the described findings and fully assess the antiparasitic efficacy of these drugs. HPLC–MS metabolite profiling of *C. coronarium* extract followed by bio-guided fractionation and isolation to purify its major components is strongly recommended in future perspectives.

## Supplementary Information

**Additional file 1.**

## Data Availability

All data generated or analyzed during this study are included in this published article.
