# Supplementary figures and images for: Repurposing drugs to treat trichinellosis: in vitro analysis of the anthelmintic activity of nifedipine and Chrysanthemum coronarium extract
Source: BMC Complement Med Ther. 2023 Jul 17;23:242. doi: 10.1186/s12906-023-04076-8 (PMC10351179; doi:10.1186/s12906-023-04076-8)

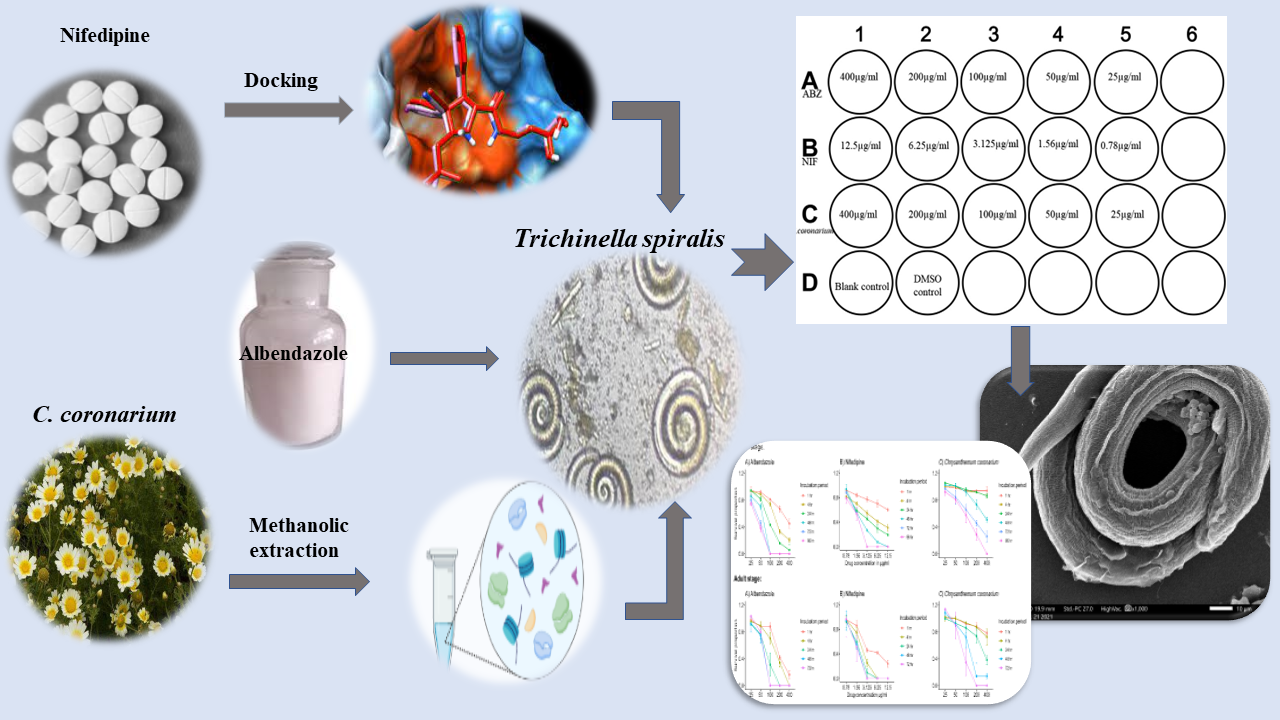

Supplement: Supplementary file 1 — Additional file 1. [file 12906_2023_4076_MOESM1_ESM.png]
